# Age distribution of dengue cases in southern Vietnam from 2000 to 2015

**DOI:** 10.1371/journal.pntd.0011137

**Published:** 2023-02-24

**Authors:** Anne-Frieda Taurel, Chan Quang Luong, Thi Thanh Thao Nguyen, Kien Quoc Do, Thanh Hai Diep, Thanh Vu Nguyen, Minh Thang Cao, Thi Nhu Dao Hoang, Phuong Thao Huynh, Thi Kim Loan Huynh, Minh Hieu Le, Joshua Nealon, Annick Moureau

**Affiliations:** 1 Sanofi, Singapore, Republic of Singapore; 2 Pasteur Institute Ho Chi Minh City, Ho Chi Minh City, Vietnam; 3 Sanofi, Lyon, France; 4 Sanofi, Marcy l’Etoile, France; Fundacao Oswaldo Cruz, BRAZIL

## Abstract

**Background:**

Dengue is the most common vector-borne viral infection. In recent times, an increase in the age of cases with clinical dengue has been reported in the national surveillance system and published literature of Vietnam. This change not only alter the risk of transmission and disease burden in different populations but also will impact for prevention and control strategies. A retrospective study was conducted from 2000 to 2015 in 19 provinces of southern Vietnam to describe the changes in age distribution of dengue cases and circulating serotypes.

**Methodology/Principal findings:**

The study is a time trend analysis of the data aggregated from the database of dengue surveillance system. The database consisted of clinically diagnosed and laboratory-confirmed cases of dengue in southern Vietnam from 2000 to 2015. In the study period, the mean age of dengue cases increased from 12.2 ± 8.8 years old (y/o) to 16.8 ± 13.3 y/o between 2000 and 2015. Majority of severe cases were observed in the age group of 5–9 y/o and 10–14 y/o. Overall, the mortality and case fatality rates (CFR) were lowest during 2010 to 2015, and all four serotypes of dengue were observed.

**Conclusions/Significance:**

With the exception of severe form, the age distribution of clinical cases of dengue appears to be shifting towards older age groups. An increase in the mean age of clinical cases of dengue has been observed in southern Vietnam over the past decade, and the highest incidence was observed in age group of 5–14 y/o. All serotypes of dengue were in circulation.

## Author summary

Dengue is associated with significant health burden in southern Vietnam. A retrospective data analysis was conducted in this study to describe the age distribution of patients with dengue during 2000–2015 to inform prevention and management strategies. Data were collected from national surveillance system to describe the patterns and changes in reported cases over the time in terms of age distribution and strains in circulation. Since 2000, the age distribution of patients confirmed with dengue has increased, and the age shift occurring mainly from 5–9 y/o to 10–14 y/o. All four dengue serotypes were observed.

## Introduction

Dengue is a mosquito-borne viral disease, primarily transmitted by *Aedes aegypti* mosquitoes (and to a lesser extent by *Ae*. *albopictus)*, *and is* caused by one of four dengue virus serotypes (DENV-1 to 4) [1]. While many infections are asymptomatic or subclinical, dengue can cause a wide array of illnesses, ranging from mild flu-like symptoms to severe and hemorrhagic dengue and, in rare cases, death [2]. The incidence rate of dengue has significantly increased in recent decades. Currently, dengue is widespread in more than 100 countries and is predominant in the Americas and Southeast Asia [2]. Approximately 75% of all these cases are estimated to occur in the Asia-Pacific region [3]. And the most commonly cited range of worldwide apparent cases is 50–100 million with around 20,000 annual deaths [4].

In Vietnam, dengue was first reported in Hanoi in the year 1958 [5]. Since 1963, the reported incidence of dengue has steadily increased until recent years [6], where dengue incidence has been highly variable, and lacked a clear trend [7]. The national case counts are mainly driven by southern Vietnam, a highly ‘dengue-endemic’ region accounting for over 85% of the total cases and deaths [8]. In recent decades, an increase in the age of patients with clinical dengue has been observed as per the data from national surveillance system and published literature [9, 10].

Studies from several countries have reported that, these changes in age distribution of dengue patients might be associated with the changes in degree of endemicity, transmission characteristics, and the circulating dengue virus strain [11–13].

The present study is the first to be conducted using data from a national surveillance system and is aimed to describe the pattern and trends in the age distribution of patients with dengue and the strains in circulation. Better understanding about the high-risk groups can help in adapting the prevention and response strategies. Recommendations can be made for the targeted high-risk groups for vaccination, once available. Additionally, the age group shift can also provide insights on the treatment and strategies to control mosquitoes.

## Methods

### Study design

**Fig 1:**
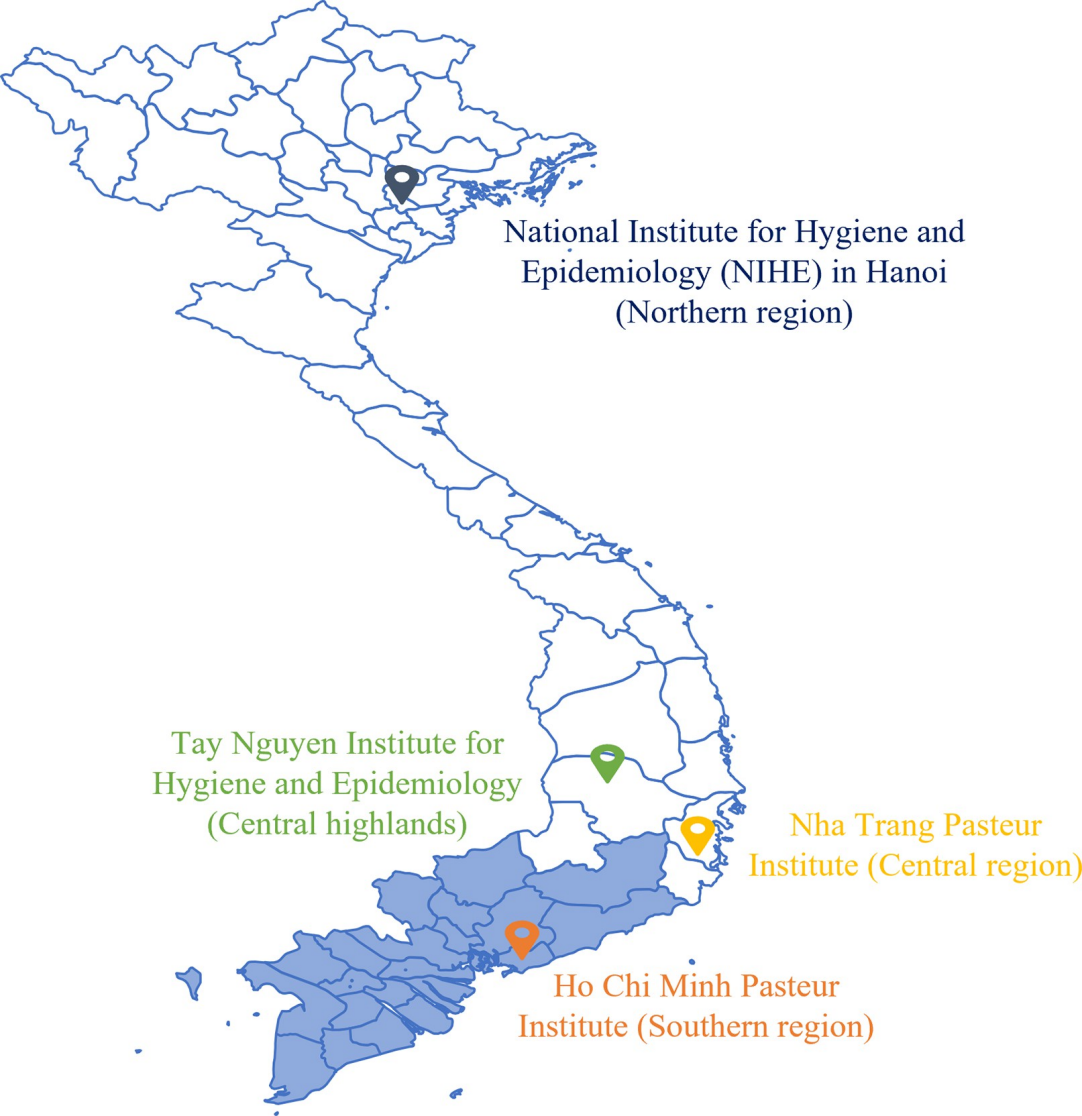
Study area– 19 provinces of southern Vietnam.

The study is a time trend analysis of the data aggregated from the dengue surveillance system database. The database comprised of clinically diagnosed and laboratory-confirmed cases of dengue from the 19 provinces of southern Vietnam between the study period of 2000 and 2015. The data was collected by Pasteur Institute of Ho Chi Minh City (PIHCMC) as a part of National Program for Dengue Control (NPDC) (**S1 Text**) (**Fig 1**) [14–17].

In 2005, to consolidate and standardize the data reporting and generating statistical reports of dengue cases, a software program named ‘CTSXH’ built on Microsoft Access was deployed in all 19 provinces of southern Vietnam by PIHCMC. All information on dengue cases was collected and entered in this program by provincial medical centers for storing and generating statistical reports. (Simplemaps.com, https://simplemaps.com/resources/svg-vn, CC0)

### Study population/data collection

All cases with a discharge diagnosis of dengue were reported in the surveillance system database. The discharge diagnoses information included patient demographics (subject’s identification, age, date of birth, sex, and province), clinical data (subject’s identification number, date of admission, date of discharge, and severity of the case), and diagnostic laboratory data (cumulative number of samples sent or tested with serology and virus isolation, polymerase chain reaction (RT-PCR), number of positive samples (serology, virus isolation, and RT-PCR), number of samples serotyped, and number of positive samples per serotype). After removal of cases with incomplete information from data collected, monthly reports were developed.

All the cases in monthly reports were categorized into ‘clinical dengue’ or ‘laboratory-confirmed dengue’. The ‘clinical dengue’ cases comprised of patients with positive diagnosis of any grade in a clinical setting. And laboratory-confirmed dengue comprised of cases with diagnosis confirmed with MAC-ELISA, virus isolation, or PCR. Both clinical dengue and laboratory-confirmed dengue cases were categorized into Grade A, B, or C, where Grade A: dengue; Grade B: dengue with warning signs; and Grade C: severe dengue. According to the MOH decision, these reported cases of dengue were divided into two groups as per their severities and the required interventions. Thus, Grade A and B were grouped in one group and Grade C in another. This second group (Grade C) consisted of cases with high risk requiring immediate intervention strategies to reduce the mortality rate.

(Case definitions are available in **S1 Text**) [15, 18].

To ensure the information extracted from the list of cases from each province was complete and accurate, the total number of cases reported in adults and children from individual listings were compared with the annual number of cases reported in the monthly reports of surveillance system. After collection of complete data, a potential bias assessment was conducted between reporting cohorts of adults and children. These reported cases were divided into two groups of ≤15 y/o and >15 y/o and the percentages of non-reported cases in adults and children were calculated. The ratios of these percentages was calculated by year for each province. The distribution of these ratios of non-reported cases in adults and children for each province was observed over time and the potential outlier provinces and/or periods of time were identified with a ratio other than 1. If this ratio was found to be other than 1, the line listings were checked for ensuring the precision. In case of an identified reporting bias, the data from the particular year and province were deleted.

The serology data was collected from PIHCMC using MAC-ELISA technique. The data was then verified by comparing the data from collection lists with periodic monthly data reports and then discrepancies in the data counts between these two sources were investigated.

Age-specific annual census data were collected from the General Statistics Office of Vietnam (GSO) survey in 1999, 2009, and 2014. For the years without age-stratified census data, the average population growth rate was calculated and applied between the surveys. E.g., for the 2015 census data, the annual population growth rate of 2009 to 2014 was used.

### Statistical analysis

The data was managed with ‘CTSXH’ program and analyzed on Microsoft Excel. The provincial mean age and standard deviation of dengue cases were calculated for each year, and Mann–Kendall test was performed for all dengue cases to detect the trend of the mean age and severity over time. Age-adjusted provincial case morbidity, mortality, and fatality rates were calculated using weighted average, and their temporal trends were described using annual percentage change (APC) for year ‘i’. The average annual percentage change (AAPC) estimates, which were the year-to-year changes in percentage and the average of year-to-year changes in percentages over the studied period ‘i’ to ‘n’ respectively:

$${\mathrm{APC}(i)}_{\mathrm{Incidence}}=\frac{I_{i}}{I_{i-1}}\times 100$$

$${\mathrm{AAPC}(i,n)}_{\mathrm{Incidence}}=\frac{\sum_{i=2}^{n}\left((I_{i}/I_{i-1}\right)-1)}{n-1}\times 100$$

where *n* is the total number of years, *I*_*i*_ is the 3-year moving average incidence rate for year *i* and *I*_*i*-1_ is the 3-year moving average incidence rate centered at the year of interest *i*-1. The same formula was applied to the mortality and CFR.

The APC was calculated yearly as the change in incidence rates from previous year (%). The AAPC (average of APC in the observed period) was assessed for the 15 years of study period and for each period of 5 years to summarize the overall yearly trends in rates during different periods of time. Annual serotype distributions were calculated from the results of laboratory-confirmed cases.

## Results

Between 2000 and 2015, southern Vietnam reported 847,929 dengue cases with an average annual incidence rate of 168 cases per 100,000 population. After data quality control, bias evaluation, and removal of incomplete data, the study contained data from: 4 provinces in the analysis set of 2000, 6 provinces in 2001, 8 provinces in 2002, 12 provinces in 2003, 13 provinces in 2004, 16 provinces in 2005, and 19 provinces between 2006 and 2015 in the analysis (**S1 Table**). The final adjusted dataset included 686,868 dengue cases from 2000 and 2015 with an overall incidence rate of 145 cases per 100,000 (13.7% reduction). A total of 92,669 laboratory-confirmed cases were present from 2000 and 2015. From these laboratory-confirmed cases, 32,572 were tested for the identification of serotypes.

**Fig 2:**
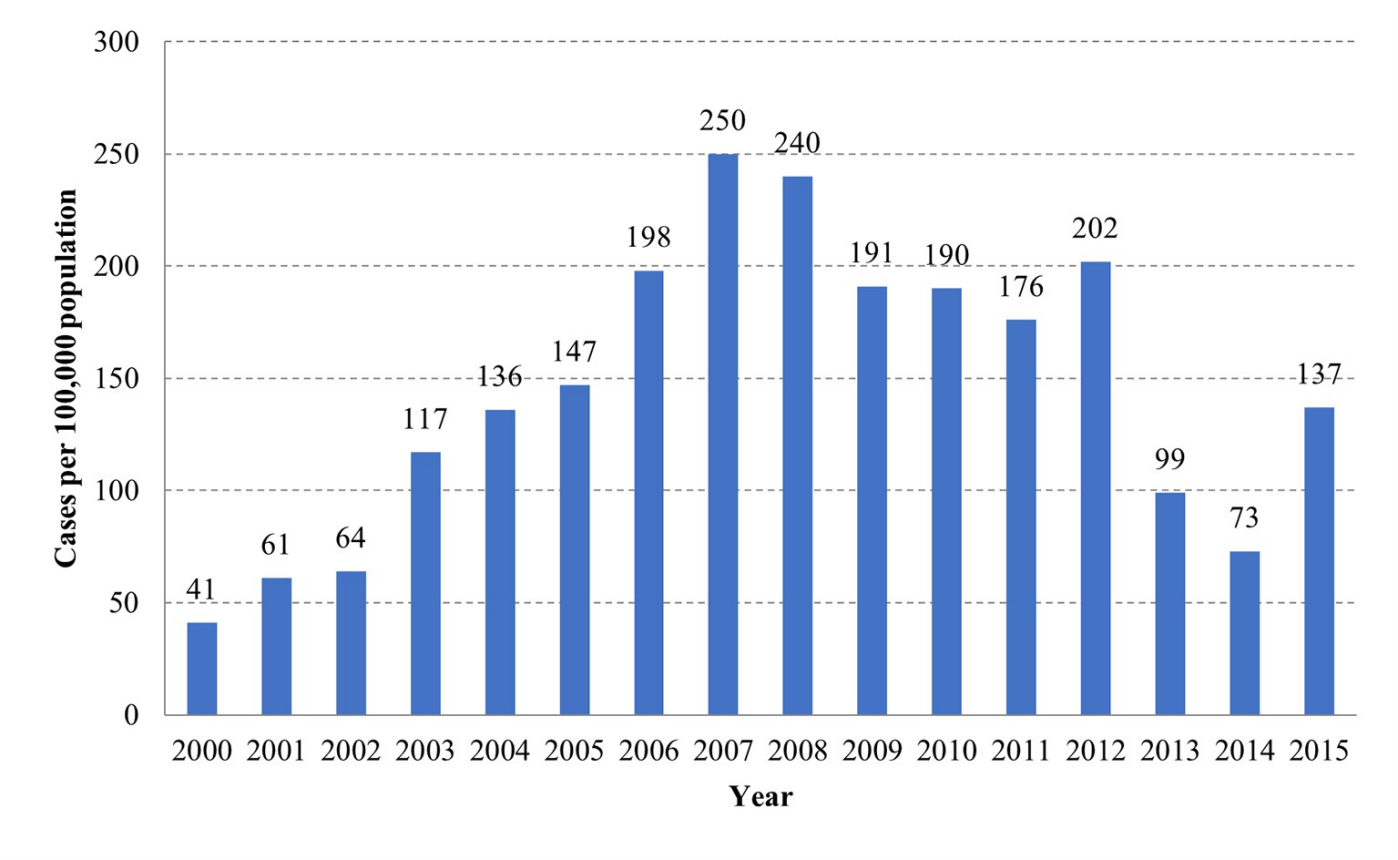
Incidence of clinical dengue cases per 100,000 population.

The incidence rate of dengue cases increased from 41 cases per 100,000 population in 2000 to 250 cases per 100,000 in 2007. From 2007 to 2015, the reported incidence rate decreased with the exception of two peaks in 2012 and 2015 (**Fig 2**). Between 2000 and 2015, the mean age of clinical dengue cases was significantly increased from 12.2 ± 8.8 years to 16.8 ± 13.3 years (**Table 1**) (Mann–Kendall test; *p* < 0.0001). Whereas the mean age of clinical dengue cases of Grade A+B (**Table 1**) **(S1 Fig)** increased from 12.4 ± 9.0 years to 17.1 ± 13.5 years (Mann-Kendall test; *p* < 0.001), and the mean age of Grade C cases showed a monotonous trend with a non-significant change from 11.1 ± 6.7 years to 12.3 ± 8.7 years (**Table 1**) **(S2 Fig)** (Mann–Kendall test; *p* = 0.24). Overall, the mean age for Grade A+B clinical dengue cases was 15.0 ± 11.3 years and 11.2 ± 7.2 years for Grade C. Throughout the study period, an increase in the mean age was observed for all grades of dengue, except for Grade C where this trend was not significant.

**Fig 3:**
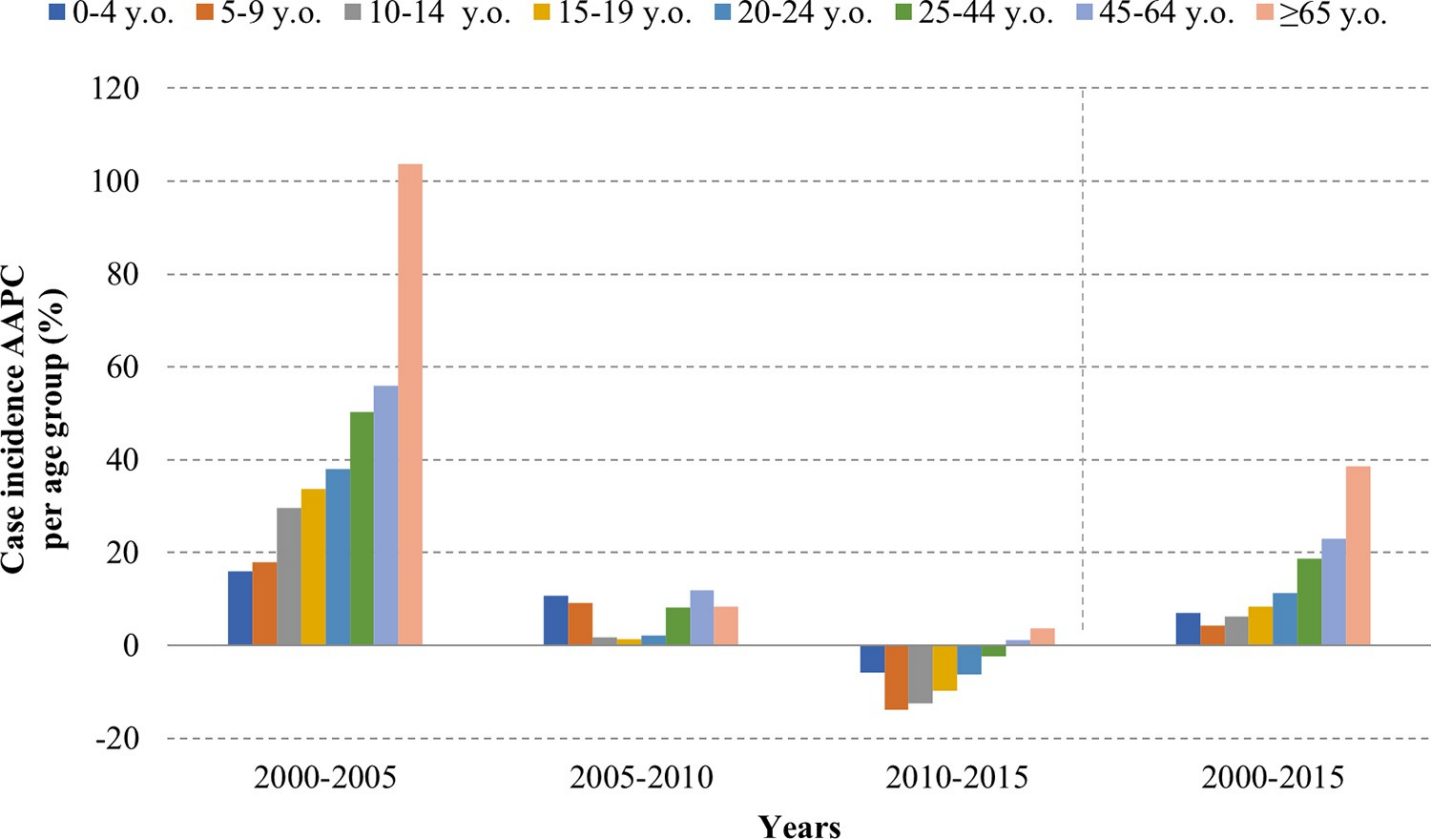
Incidence of clinical dengue cases using average annual percent change in reported dengue case incidence per age group.

The incidence rate of dengue was found to be non-monotonic. The incidence rate was found to be increasing from year 2000 and then peaked in 2007. This peak was followed by a decline in incidence rates except for 2012 and 2015 where the incidence rates were again higher the previous years. The AAPC for incidence rate showed an increasing trend in all age groups. This increase was found to be increasing with the age and ranged from 4.4% in 5–9 y/o to 38.7% in >65 y/o. Similarly, for reported cases, the AAPC was found to be increasing throughout the study period, and especially between the 2000 to 2005 period (**Fig 3**). This increase was driven by high AAPC values which ranged from 16% to 103% per age group between 2000 and 2005. From 2005 to 2010, the AAPC showed a limited surge, which was 1.3% in the 15–19 y/o age group to 11.8% the 45–64 y/o group in 2010. From 2010 to 2015, all age groups <45 y/o exhibited a decreasing incidence trend with AAPC ranging from -13.9% to -2.4%. The groups of 45–65 y/o and >65 y/o exhibited a limited but increasing incidence trend of 1.2% and 3.8%, respectively.

**Fig 4:**
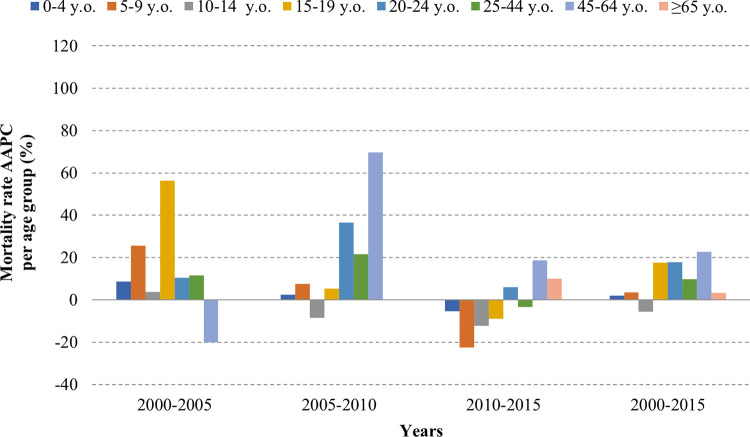
Average annual percent change in dengue mortality per age group.

**Fig 5:**
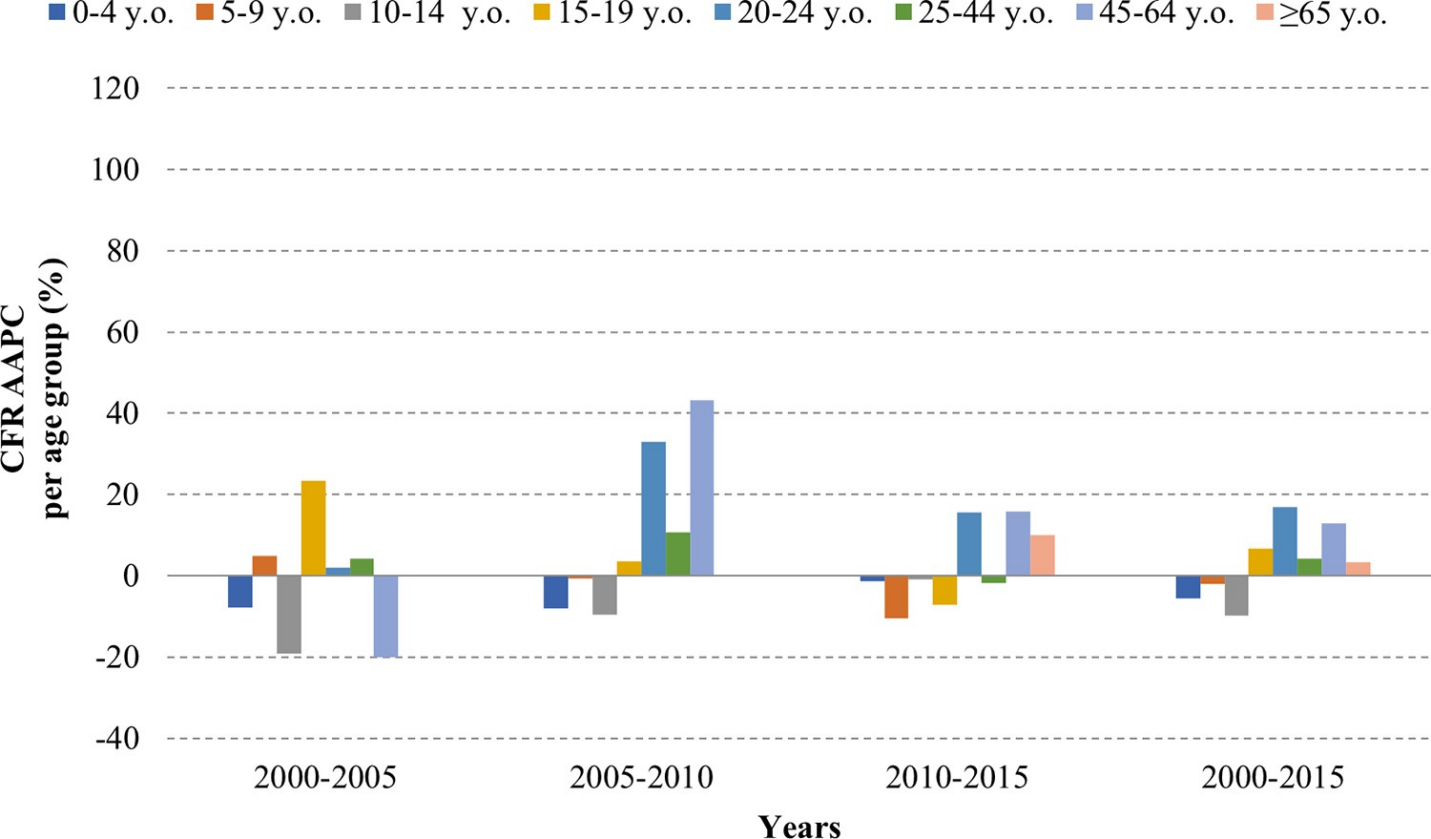
Average annual percent change in dengue case fatality rate per age group.

In contrast, the AAPC showed a heterogeneous trend for mortality and CFR over the time and age groups. While a steady increase was observed in group of 15–64 y/o, a decrease or a limited increase was observed in the groups of <15 y/o and ≥65 y/o. (**Figs 3, 4 and 5**)

**Fig 6:**
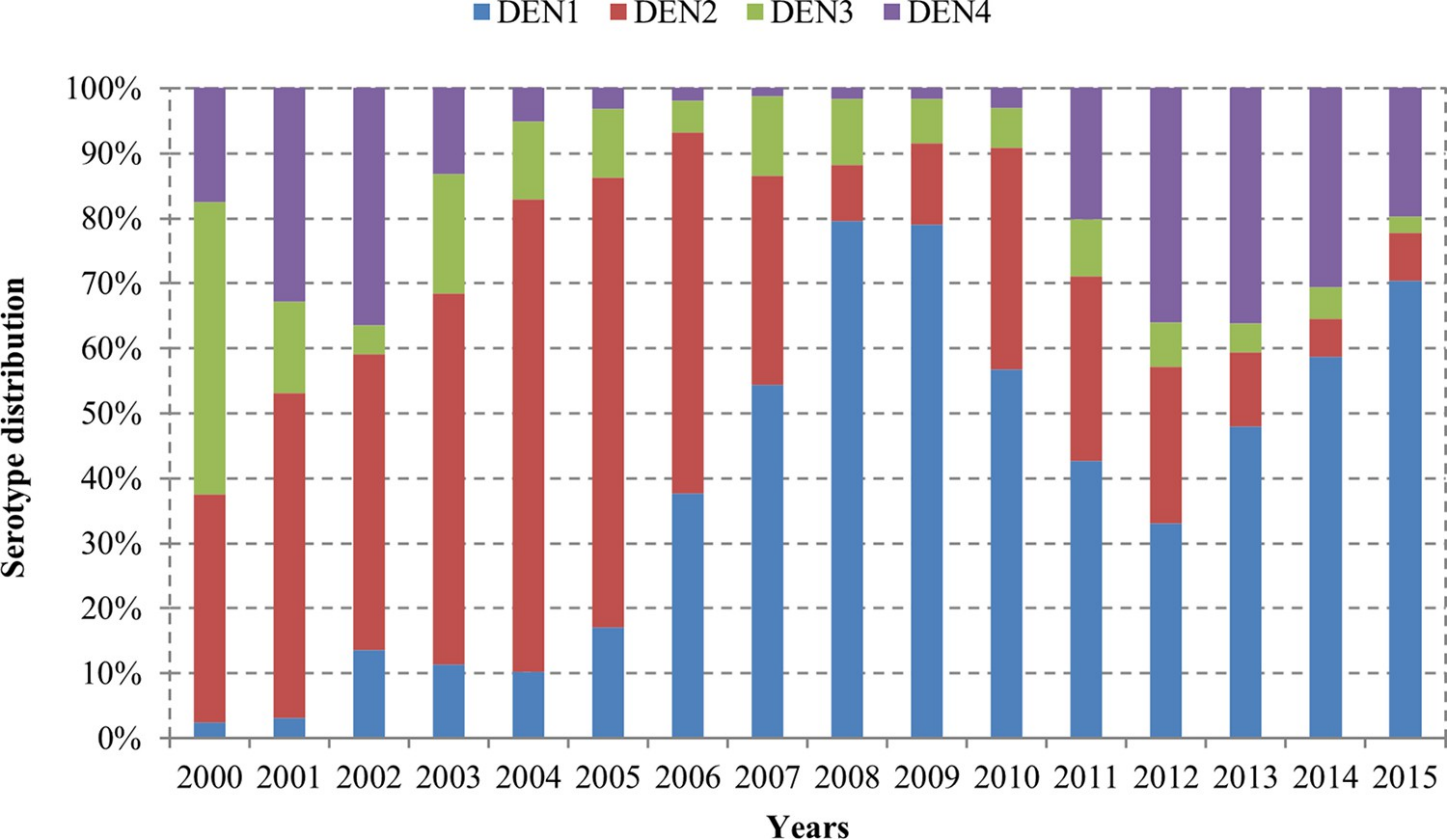
Annual distribution of dengue serotypes (N = 32,572).

All four serotypes of dengue were circulating in southern Vietnam. During the study period, the predominant serotype switched four times; from DENV-3 to DENV-2 (2001), DENV-2 to DENV-1 (2007), DENV-1 to DENV-4 (2012), and DENV-4 to DENV-1 (2013). Overall, DENV-2 and DENV-1 were the main circulating serotypes. DENV-2 was the predominant serotype during 2001–2006 and represented 45%–72% of all serotypes in circulation. Whereas DENV-1 represented 33%–79% during 2007–2015. Except for 2012, when DENV-4 was the predominant serotype, and represented 36% of all. (**Fig 6**). This switch of predominant serotypes was followed by the dynamics of reported case incidence rates and age distribution (**Fig 2**).

**Table 1:** Age distribution trend of clinical dengue cases, dengue cases and dengue with warning signs (Grade A+B), and severe dengue (Grade C).

	Age distribution trend of clinical dengue cases					
	Clinical dengue cases		Dengue cases and dengue with warning signs (Grade A+B)		Severe dengue (Grade C)	
Year	Mean age in years (SD)	Total no. of cases	Mean age in years (SD)	Total no. of cases	Mean age in years (SD)	Total no. of cases
2000	12.2 (8.8)	3,431	12.4 (9.0)	3,090	11.1 (6.7)	341
2001	11.6 (8.3)	7,272	11.8 (8.6)	6,244	10.7 (6.1)	1,028
2002	13.1 (8.9)	9,556	13.3 (9.2)	8,366	11.3 (6.1)	1,190
2003	13.3 (8.3)	23,121	13.5 (8.6)	19,508	11.5 (6.0)	3,613
2004	13.1 (9.4)	30,450	13.3 (9.6)	25,470	11.9 (8.1)	4,980
2005	14.4 (9.0)	39,305	14.6 (9.3)	33,314	11.8 (6.5)	5,991
2006	14.1 (9.4)	61,247	14.5 (9.7)	51,980	11.9 (7.2)	9,267
2007	13.4 (9.3)	78,382	13.7 (9.5)	68,504	11.3 (7.3)	9,878
2008	13.3 (9.8)	76,704	13.7 (10.1)	67,348	10.2 (6.3)	9,356
2009	13.5 (9.9)	61,873	13.9 (10.2)	53,759	10.3 (6.4)	8,114
2010	14.3 (10.9)	62,321	14.8 (11.2)	54,441	10.6 (7.0)	7,880
2011	15.8 (11.9)	58,647	16.5 (12.2)	51,739	10.7 (7.4)	6,908
2012	16.6 (13.1)	68,102	17.2 (13.4)	62,225	11.2 (8.5)	5,877
2013	17.3 (13.8)	33,702	17.7 (14.0)	31,403	11.9 (9.1)	2,299
2014	17.0 (14.0)	25,023	17.2 (14.1)	23,678	12.7 (9.3)	1,345
2015	16.8 (13.3)	47,732	17.1 (13.5)	44,896	12.3 (8.7)	2,836
Avg. mean age (SD) and Total no of cases	**14.5 (10.9)**	**686,868**	**15.0 (11.3)**	**605,965**	**11.2 (7.2)**	**80,903**
Mann-Kendall Test	***Sen’s slope*:** 0.33; p < 0.0001		***Sen’s slope*:** 0.361; p < 0.001		***Sen’s slope*:** 0.072; p = 0.239	

Abbreviation: SD, standard deviation.

## Discussion

Before 2000s, the age distribution dengue cases were essentially under 15 y/o in the southern Vietnam. Particularly during 1978–1992, the number of patients suffering from dengue hemorrhagic fever essentially consisted of children under 9 y/o. The age group with highest morbidity was 5–6 y/o, whereas fewer cases occurred in adults [19]. Similarly in 1998 and 1999, the cases of dengue fever and dengue hemorrhagic fever mostly occurred in children under 15 y/o which accounted for 90% of the total cases [20].

Age is an important risk factor associated with the occurrence and severity of symptomatic dengue and younger children are more likely to have severe clinical course [21]. As evident from current and aforenamed studies, with this shift of mean age of dengue cases from young children to adolescents and adults, it is very possible that dengue may not remain exclusive to the younger population. This age shift will necessitate an update in current epidemiological surveillance, control, prevention and management strategies focusing on a wider population covering children as well as adults. These findings will be helpful to the health care systems in regions with most vulnerable population and dengue is an endemic. So that the healthcare system can plan, train, and educate people to foster awareness about dengue.

Our findings are similar to the observations from previous studies conducted in dengue endemic countries, such as Indonesia and Thailand where a change in age distribution to older age has been observed [22, 23]. Additionally, several serology surveys conducted in Vietnam indicated that dengue cases have shifted to older age groups [24]. A study by Quang et al. showed that the median age of dengue cases is increasing. During 2006 to 2010, 56.8% of cases were ≤15 years of age, whereas in 2013, they were 43.6% of the total cases occurred [16]. The increase in AAPC with the age group could be associated with the changes in the health-seeking behaviors of and/or true changes in the age distribution of patients with dengue.

Our findings indicate that all four serotypes were in circulation in southern Vietnam between 2000 and 2015. We observed, the clinical dengue cases peaked with the switch of predominant serotype. These changes in serotype distribution were reflected in the changes of reported case incidence rates and age distribution. Both observations could be associated with an increased immunity in the population, i.e., the peak may correlate with a switch in circulating serotypes which was associated with a rise in secondary infections and symptomatic cases linked to new serotype. Additional factors associated with the serotype characteristics may impact the virulence or transmission [25, 26]. The descriptive nature of our study cannot address these hypotheses, necessitating additional investigation on the association of dengue virus serotypes and genotypes with outbreak characteristics.

Our study has some limitations. First, the retrospective nature of study design may have contributed to incompleteness of data due to potential issues with data reporting, especially before 2005. Second, this is a purely descriptive analysis, not allowing discussion on the possible association between age and epidemiological characteristics of dengue in Vietnam. Lastly, these results are not representative of entire Vietnam as the data were analyzed for the southern region only.

## Conclusion

With the exception of its severe form, the age distribution of the clinical cases of dengue in southern Vietnam appears to be shifting towards older age groups. The mean age of dengue cases has significantly increased with the 10–15 y/o age group reporting the highest incidence in 2015 *vs*. 5–9 y/o in 2000. Over last 15 years, the morbidity, mortality, and CFR of dengue have decreased in younger children and adolescents (age group <15 y/o) and increased in adults and older adults. With this shift in age distribution of dengue, the strategies of interventions, communications, and risk assessment also need to be updated. Better understanding of these age distribution patterns would enable in predicting epidemiological patterns and setting up vaccination strategies.

Being a major public health concern, the control and prevention of dengue would be key to mitigate its burden. Additional studies will be required to identify other factors associated with the changes in the age distribution of patients with dengue.

## Supporting information

S1 Text Dengue surveillance system in Vietnam and case definitions of clinical dengue cases classification. (DOCX)

S1 Table Data quality check results of annual provincial dengue case reports in South of Vietnam, 2000–2015. (DOCX)

S1 Fig Incidence of dengue cases per 100,000 population per age group by Grade A+B. (TIF)

S2 Fig Incidence of dengue cases per 100,000 population per age group by Grade C. (TIF)
