# Supplementary material for: Age distribution of dengue cases in southern Vietnam from 2000 to 2015
Source: PLoS Negl Trop Dis. 2023 Feb 24;17(2):e0011137. doi: 10.1371/journal.pntd.0011137 (PMC9994699; doi:10.1371/journal.pntd.0011137)
Supplement: S1 Text — (DOCX) [file pntd.0011137.s001.docx]

**Supporting information**

**Dengue surveillance system in Vietnam**

Following a severe dengue outbreak in 1998, the National Program for Dengue Control (NPDC) was established in 1999 with an aim to reduce the morbidity and mortality due to dengue. This NPDC conducts epidemiological surveillance and is supported by four regional institutes that provide the technical and laboratory support: The National Institute for Hygiene and Epidemiology (NIHE) in Hanoi for the northern region, the Tay Nguyen Institute for Hygiene and Epidemiology for the highlands, Pasteur Institute of Nha Trang for the central region, and the Pasteur Institute of Ho Chi Minh City (PIHCMC) for the southern Vietnam. The surveillance system is designed to detect dengue cases at community health service level in a timely and standardized manner.

*Reporting of dengue cases to surveillance system*

All cases of clinical dengue from all clinical settings (regional hospitals, provincial hospitals, district health centers, public and private clinics) were included in the surveillance system. Line listing of dengue was made by tracing the case information during an episode of illness. From these line listings, duplications were eliminated, and the data was modified/corrected as necessary. From this quality-controlled data the monthly reports were generated.

**Case definitions of clinical dengue classification**

Until 2011, the case definition of dengue was based upon four levels of staging diagnosis, e.g., Stage I, II, III, and IV, and then it switched to the World Health Organization’s 2009 diagnostic criteria, which was based upon in three stages: dengue, dengue with warning signs and severe dengue. This criterion provided information on the severity by age (≤15 y/o and >15 y/o) and the serotypes in circulation.

*Dengue (grade A):*

- Patient has recently travelled to or lives in an endemic area, has a fever or two of the following: nausea or vomiting, rash, aches and pain, positive torniquet test, leukopaenia and any warning sign
- Laboratory-confirmed dengue virus infection

*Dengue with warning signs (grade B):*

- Abdominal pain or tenderness
- Persistent vomiting
- Clinical fluid accumulation
- Mucosal bleeding
- Lethargy or restlessness
- Liver enlargement of >2 cm
- An increases haematocrit level that is accompainied by a decreased platelet count

*Severe dengue (grade C):*

- Severe plasma leakage leading to dengue shock syndrome and fluid accumulation with respiratory distress
- Severe bleeding as evaluated by a clinician
- Severe organ involvement, including the liver (AST or ALT levels of ≥1.000 UI/ml), the central nervous system (impaired consciousness), the heart and other organs
