# Supplementary material for: Age distribution of dengue cases in southern Vietnam from 2000 to 2015
Source: PLoS Negl Trop Dis. 2023 Feb 24;17(2):e0011137. doi: 10.1371/journal.pntd.0011137 (PMC9994699; doi:10.1371/journal.pntd.0011137)
Supplement: S1 Table — (DOCX) [file pntd.0011137.s002.docx]

**S1 Table. Data quality check results of annual provincial dengue case reports in South of Vietnam, 2000-2015**

Clinical cases

| **Year** | **S1** | **S2** | **S3** | **S4** | **S5** | **S6** | **S7** | **S8** | **S9** | **S10** | **S11** | **S12** | **S13** | **S14** | **S15** | **S16** | **S17** | **S18** | **S19** | **No. of provinces with data** |
| --- | --- | --- | --- | --- | --- | --- | --- | --- | --- | --- | --- | --- | --- | --- | --- | --- | --- | --- | --- | --- |
| **2000** | - | - | Y | Y | - | - | - | Y | - | - | - | - | - | - | - | - | Y | - | - | **4** |
| **2001** | - | - | Y | Y | - | - | Y | Y | - | - | - | - | Y | - | - | - | Y | - | - | **6** |
| **2002** | - | - | Y | Y | - | - | Y | Y | - | - | - | Y | Y | - | - | Y | Y | - | - | **8** |
| **2003** | - | Y | Y | Y | - | Y | Y | Y | - | Y | - | Y | Y | Y | - | Y | Y | - | - | **12** |
| **2004** | - | Y | Y | Y | - | Y | Y | Y | Y | Y | - | Y | Y | Y | - | Y | Y | - | - | **13** |
| **2005** | Y | Y | Y | Y | Y | Y | Y | Y | Y | Y | - | Y | Y | Y | - | Y | Y | - | Y | **16** |
| **2006** | Y | Y | Y | Y | Y | Y | Y | Y | Y | Y | Y | Y | Y | Y | Y | Y | Y | Y | Y | **19** |
| **2007** | Y | Y | Y | Y | Y | Y | Y | Y | Y | Y | Y | Y | Y | Y | Y | Y | Y | Y | Y | **19** |
| **2008** | Y | Y | Y | Y | Y | Y | Y | Y | Y | Y | Y | Y | Y | Y | Y | Y | Y | Y | Y | **19** |
| **2009** | Y | Y | Y | Y | Y | Y | Y | Y | Y | Y | Y | Y | Y | Y | Y | Y | Y | Y | Y | **19** |
| **2010** | Y | Y | Y | Y | Y | Y | Y | Y | Y | Y | Y | Y | Y | Y | Y | Y | Y | Y | Y | **19** |
| **2011** | Y | Y | Y | Y | Y | Y | Y | Y | Y | Y | Y | Y | Y | Y | Y | Y | Y | Y | Y | **19** |
| **2012** | Y | Y | Y | Y | Y | Y | Y | Y | Y | Y | Y | Y | Y | Y | Y | Y | Y | Y | Y | **19** |
| **2013** | Y | Y | Y | Y | Y | Y | Y | Y | Y | Y | Y | Y | Y | Y | Y | Y | Y | Y | Y | **19** |
| **2014** | Y | Y | Y | Y | Y | Y | Y | Y | Y | Y | Y | Y | Y | Y | Y | Y | Y | Y | Y | **19** |
| **2015** | Y | Y | Y | Y | Y | Y | Y | Y | Y | Y | Y | Y | Y | Y | Y | Y | Y | Y | Y | **19** |

Laboratory cases

| **Year** | **S1** | **S2** | **S3** | **S4** | **S5** | **S6** | **S7** | **S8** | **S9** | **S10** | **S11** | **S12** | **S13** | **S14** | **S15** | **S16** | **S17** | **S18** | **S19** | **No. of provinces with data** |
| --- | --- | --- | --- | --- | --- | --- | --- | --- | --- | --- | --- | --- | --- | --- | --- | --- | --- | --- | --- | --- |
| **2000** | Y | Y | Y | Y | Y | Y | Y | Y | Y | Y | Y | Y | Y | Y | Y | Y | Y | Y | Y | **19** |
| **2001** | Y | Y | Y | Y | Y | Y | Y | Y | Y | Y | Y | Y | Y | Y | Y | Y | Y | Y | Y | **19** |
| **2002** | Y | Y | Y | Y | Y | Y | Y | Y | Y | Y | Y | Y | Y | Y | Y | Y | Y | Y | Y | **19** |
| **2003** | Y | Y | Y | Y | Y | Y | Y | Y | Y | Y | Y | Y | Y | Y | Y | Y | Y | Y | Y | **19** |
| **2004** | Y | Y | Y | Y | Y | Y | Y | Y | Y | Y | Y | Y | Y | Y | Y | Y | Y | Y | Y | **19** |
| **2005** | Y | Y | Y | Y | Y | Y | Y | Y | Y | Y | Y | Y | Y | Y | Y | Y | Y | Y | Y | **19** |
| **2006** | Y | Y | Y | Y | Y | Y | Y | Y | Y | Y | Y | Y | Y | Y | Y | Y | Y | Y | Y | **19** |
| **2007** | Y | Y | Y | Y | Y | Y | Y | Y | Y | Y | Y | Y | Y | Y | Y | Y | Y | Y | Y | **19** |
| **2008** | Y | Y | Y | Y | Y | Y | Y | Y | Y | Y | Y | Y | Y | Y | Y | Y | Y | Y | Y | **19** |
| **2009** | Y | Y | Y | Y | Y | Y | Y | Y | Y | Y | Y | Y | Y | Y | Y | Y | Y | Y | Y | **19** |
| **2010** | Y | Y | Y | Y | Y | Y | Y | Y | Y | Y | Y | Y | Y | Y | Y | Y | Y | Y | Y | **19** |
| **2011** | Y | Y | Y | Y | Y | Y | Y | Y | Y | Y | Y | Y | Y | Y | Y | Y | Y | Y | Y | **19** |
| **2012** | Y | Y | Y | Y | Y | Y | Y | Y | Y | Y | Y | Y | Y | Y | Y | Y | Y | Y | Y | **19** |
| **2013** | Y | Y | Y | Y | Y | Y | Y | Y | Y | Y | Y | Y | Y | Y | Y | Y | Y | Y | Y | **19** |
| **2014** | Y | Y | Y | Y | Y | Y | Y | Y | Y | Y | Y | Y | Y | Y | Y | Y | Y | Y | Y | **19** |
| **2015** | Y | Y | Y | Y | Y | Y | Y | Y | Y | Y | Y | Y | Y | Y | Y | Y | Y | Y | Y | **19** |
