# Supplementary figures and images for: Age distribution of dengue cases in southern Vietnam from 2000 to 2015
Source: PLoS Negl Trop Dis. 2023 Feb 24;17(2):e0011137. doi: 10.1371/journal.pntd.0011137 (PMC9994699; doi:10.1371/journal.pntd.0011137)

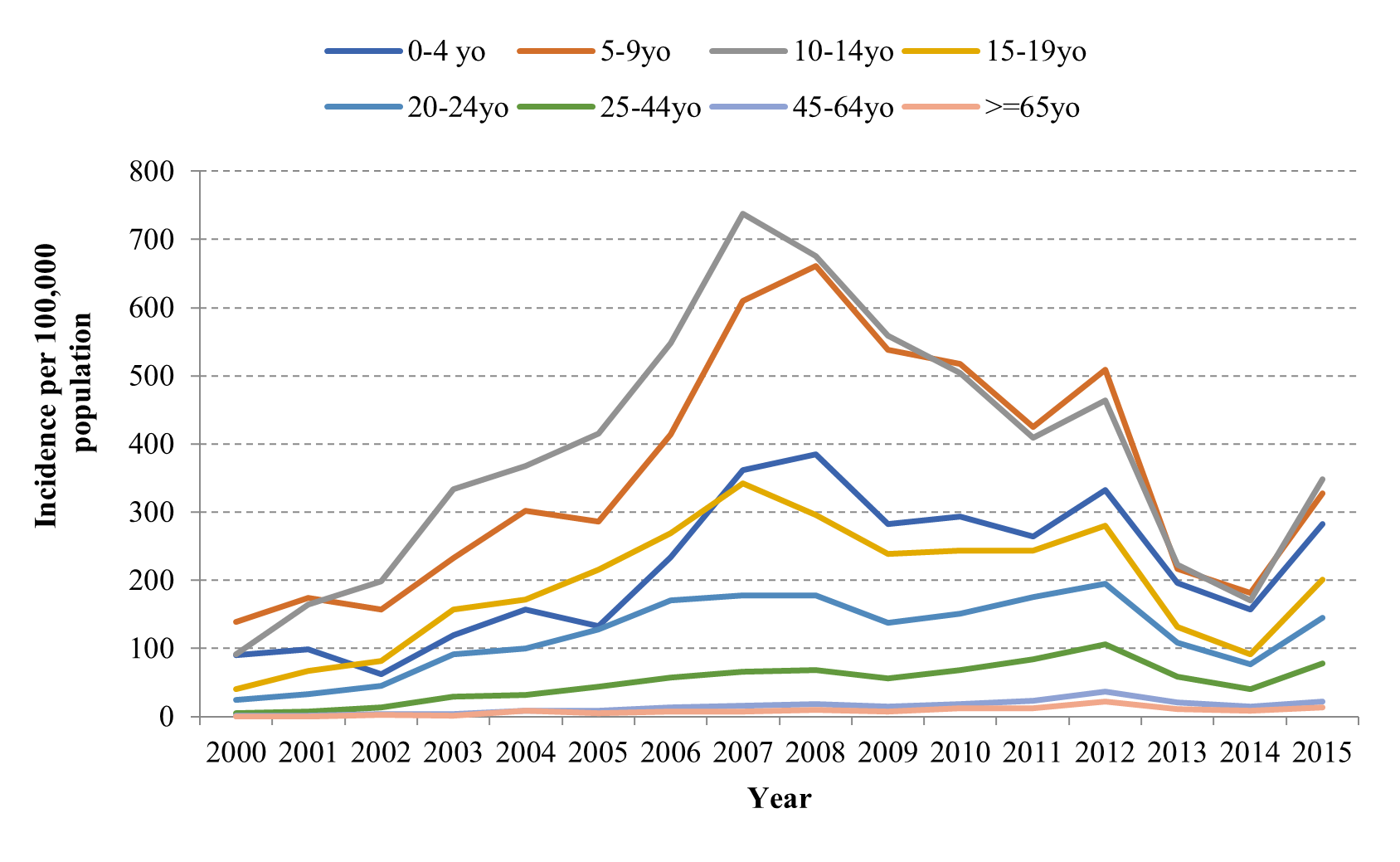

Supplement: S1 Fig — (TIF) [file pntd.0011137.s003.tif]

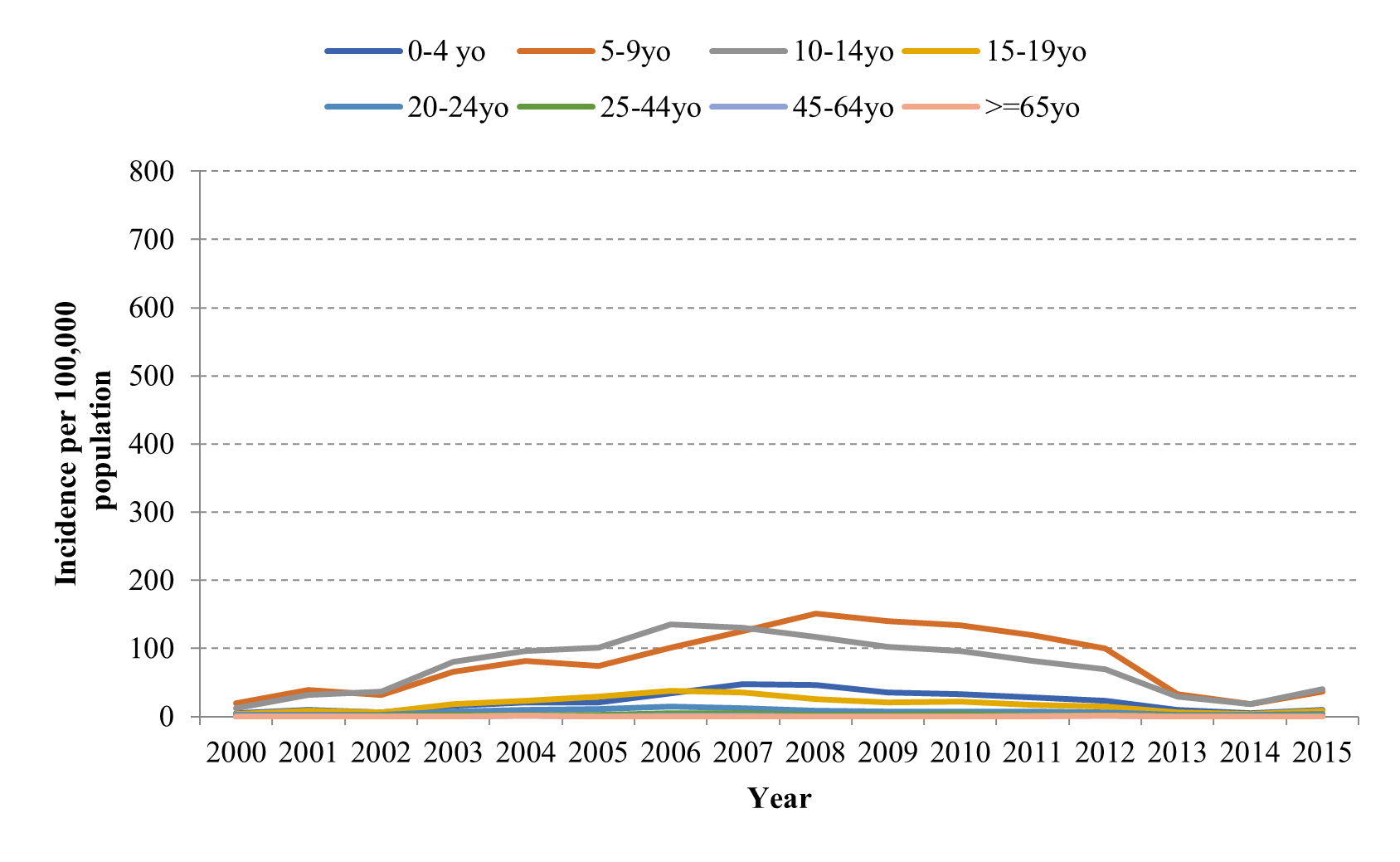

Supplement: S2 Fig — (TIF) [file pntd.0011137.s004.tif]
